# TAS2R20 variants confer dietary adaptation to high‐quercitrin bamboo leaves in Qinling giant pandas

**DOI:** 10.1002/ece3.6327

**Published:** 2020-05-04

**Authors:** Xiangxu Hu, Guan Wang, Lei Shan, Shuyan Sun, Yibo Hu, Fuwen Wei

**Affiliations:** ^1^ Key Laboratory of Animal Ecology and Conservation Biology Institute of Zoology Chinese Academy of Sciences Beijing China; ^2^ University of Chinese Academy of Sciences Beijing China; ^3^ Department of Laboratory Medicine Boston Children’s Hospital and Harvard Medical School Boston MA USA; ^4^ Jiangsu Key Laboratory for Biodiversity and Biotechnology College of Life Sciences Nanjing Normal University Nanjing China; ^5^ Center for Excellence in Animal Evolution and Genetics Chinese Academy of Sciences Kunming China

**Keywords:** bamboo leaves, bitter taste receptor, dietary adaptation, giant panda, quercitrin

## Abstract

Sensitivity to bitter tastes provides animals with an important means of interacting with their environment and thus, influences their dietary preferences. Genetic variants encoding functionally distinct receptor types contribute to variation in bitter taste sensitivity. Our previous study showed that two nonsynonymous sites, A52V and Q296H, in the *TAS2R20* gene are directionally selected in giant pandas from the Qinling Mountains, which are speculated to be the causative base‐pair changes of Qinling pandas for the higher preference for bamboo leaves in comparison with other pandas. Here, we used functional expression in engineered cells to identify agonists of pTAS2R20 (i.e., giant panda's TAS2R20) and interrogated the differences in perception in the in vitro responses of pTAS2R20 variants to the agonists. Our results show that pTAS2R20 is specifically activated by quercitrin and that pTAS2R20 variants exhibit differences in the sensitivity of their response to the agonist. Compared with pTAS2R20 in pandas from other areas, the receptor variant with A52V and Q296H, which is most commonly found in Qinling pandas, confers a significantly decreased sensitivity to quercitrin. We subsequently quantified the quercitrin content of the leaves of bamboo distributed in the Qinling Mountains, which was found to be significantly higher than that of the leaves of bamboo from panda habitats in other areas. Our results suggest that the decreased sensitivity to quercitrin in Qinling pandas results in higher‐quercitrin‐containing bamboo leaves to be tasting less bitter to them and thus, influences their dietary preference. This study illustrates the genetic adaptation of Qinling pandas to their environments and provides a fine example of the functional effects of directional selection in the giant panda.

## Graphical abstract

Sensitivity to bitter tastes provides animals with an important means of interacting with their environment and thus, influences their dietary preferences. TAS2R20 variants confer dietary adaptation to high‐quercitrin bamboo leaves in Qinling giant pandas. The decreased sensitivity to quercitrin in Qinling pandas results in higher‐quercitrin‐containing bamboo leaves to be tasting less bitter to them and thus, influences their dietary preference.

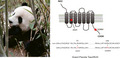

## 1 INTRODUCTION

Animals taste receptor genes evolve in response to species‐specific diets (Jiang et al., 2012; Li et al., 2005; Liu et al., 2016; Sato & Wolsan, 2012; Shan, Wu, Wang, Zhang, & Wei, 2018; Zhao, Yang, Xu, & Zhang, 2010). Food‐derived compounds stimulate taste receptors on specialized cells within taste buds, causing pleasant or unpleasant sensations and, thus, influencing the dietary preferences of animals (Breslin, 2013; Chandrashekar, Hoon, Ryba, & Zuker, 2006; Fujikura, 2015). Among the possible taste sensations, bitter taste, mediated by bitter taste 2 receptors (TAS2Rs), is viewed as a defense system of animals against toxic compounds in food, as toxins usually taste bitter and trigger aversive reactions. In nature, there are a wide variety of bitter substances such as plant alkaloids, most of which may be toxic and harmful (Lossow et al., 2016). Given the ubiquitous bitterness that exists in the environment that species occupy, animals harbor a family of receptors that are responsive to bitter compounds (Bufe, Hofmann, Krautwurst, Raguse, & Meyerhof, 2002), and each TAS2R is commonly responsive to several bitter compounds (Meyerhof et al., 2010). Studies have demonstrated that the numbers of TAS2R vary greatly among species from a few to tens of these receptors, and they have been lost and gained frequently in the course of animal evolution (Hayakawa, Suzuki‐Hashido, Matsui, & Go, 2014; Li & Zhang, 2013; Liu et al., 2016). A correlation has been revealed between the TAS2R number in a species and the fraction of plants in its diet, because plant tissues contain more toxic compounds than animal tissues, indicating that dietary toxins are a major selective force shaping the evolution of TAS2Rs (Li & Zhang, 2013). In addition, it has been reported that the nonsynonymous K172N site in the human bitter receptor TAS2R16 gene was associated with an increased sensitivity to salicin, arbutin, and five different cyanogenic glycosides (Soranzo et al., 2005); sequence variants in the TAS2R38 gene are correlated with variable taste sensitivity to the bitter compound phenylthiocarbamide (PTC) in humans, chimpanzees, and macaques (Suzuki et al., 2010; Wooding et al., 2006). These reports suggested that receptor variants could result in different sensitivities to the same bitter compounds, leading to species‐ and/or population‐level differences in dietary preferences, reflecting the potential roles of these variants in the transformation of animal feeding and dietary adaptation (Bufe et al., 2005; Nei, Niimura, & Nozawa, 2008; Soranzo et al., 2005; Wooding et al., 2006).

The giant panda (*Ailuropoda melanoleuca*) is a specialized herbivore in the order Carnivora that feeds almost exclusively on highly fibrous bamboo and exhibits a mix of herbivore and carnivore traits: It has obligate bamboo diet but retains a carnivore‐typical digestive tract. As a result of this dietary specialization, a series of adaptations to bamboo can be observed in panda morphology, behavior, physiology, genetics, and their intestinal microbiome (Hu et al., 2017; Nie, Speakman, et al., 2015; Nie et al., 2019; Nie, Zhang, et al., 2015; Wei et al., 2014; Wei, Swaisgood, et al., 2015; Wei, Wang, & Wu, 2015; Wei et al., 2019; Zhou et al., 2019; Zhu, Wu, Dai, Zhang, & Wei, 2011). Paleontological and molecular evidence suggests that ancient pandas were carnivorous or omnivorous and that they switched to a bamboo diet at least ∼7.0 million years ago (Jin et al., 2007; Zhao et al., 2010). However, a recent report based on stable isotope analyses suggests that ancient pandas may have had more complex diets than modern pandas (Han et al., 2019).

Multiple lines of evidences indicate that plants have been components of pandas’ diet for several million years, and this type of diet poses the challenge of tolerating large amounts of bitter compounds. Our previous work showed that there are more putatively functional TAS2Rs in the giant panda than in other carnivores, which might be expected because the abundant bitter substances encountered by pandas could lead to a requirement for more functional TAS2Rs for bitter taste perception (Shan et al., 2018). The purifying selection pressure on three TAS2R genes (*TAS2R1*, *9*, and *38*) is markedly strengthened in the species, suggesting that these three receptor gene sequences are specifically highly conserved, probably because of the presence of some sites that are functionally more important for the detection of certain bitter compounds in the panda diet. Additionally, signatures of positive selection were detected for *TAS2R42* and *TAS2R49* in pandas. *TAS2R49* is now designated as *TAS2R20* according to the last Gene Nomenclature Committee of the Human Genome Organization (http://www.genenames.org/, last accessed April 30, 2016), and this gene has been directionally selected at two nonsynonymous sites A52V and Q296H in the panda population from the Qinling Mountains (Qinling pandas) (Shan et al., 2018; Zhao et al., 2013). Consistent with this finding, field observations showed that Qinling pandas consume more bamboo leaves than pandas in other areas (Pan et al., 2001; Schaller, Hu, Pan, & Zhu, 1985); population genetic data indicated their divergence from other pandas ~0.3 million years ago and showed genetic adaptation to their environments (Wei et al., 2014; Zhao et al., 2013). These findings collectively raise the question of whether the two nonsynonymous sites in *TAS2R20* are the causative base‐pair changes resulting in the preference of Qinling pandas for the consumption of more bamboo leaves than the pandas from other areas. We hypothesized that the two nonsynonymous sites in *TAS2R20* encode receptor variants that may decrease Qinling pandas’ taste sensitivity to bitter compounds, causing bamboo leaves to taste less bitter to the pandas.

To address this hypothesis, we first challenged pTAS2R20 with several common bitter substances (caffeine, sesquiterpene lactone, denatonium benzoate, chloroquine, picrotoxinin, cycloheximide, and nicotine), and some known bamboo‐derived bitter chemicals (quercitrin, tannin, salicin, aloin, coumarin, amygdalin, and galangin) in a heterologous expression system. Among these bitter compounds, pTAS2R20 was specifically activated by quercitrin, a flavonoid monomer found in various plants including bamboos. Then, we used high‐performance liquid chromatography (HPLC) to quantify the quercitrin contents of the leaves of *Bashania fargesii* and *Fargesia qinlingensis* for which Qinling pandas show the strongest preference, and compared the results with the quercitrin contents of the leaves of *Fargesia denudata* and *Bshania faberi*, which other pandas prefer. Finally, four pTAS2R20 variants were generated and challenged with quercitrin. The two significantly selected nonsynonymous sites in *TAS2R20* occur at amino acid position 52, where either an alanine or a valine is encoded, and position 296, where either a glutamine or a histidine is encoded, giving rise to AQ, VQ, AH, and VH receptor variants. In nature, only two haplotypes VH and AQ are found in Qinling pandas and pandas from other areas, respectively, whereas VQ and AH are mutated variants used for examining the effect of each of the two nonsynonymous sites on the function of pTAS2R20 in response to its agonist. By combining these strategies, we expected to verify the hypothesis, and reveal how polymorphisms in p*TAS2R20* influence quercitrin perception in giant pandas, providing an example of the functional effects of directional selection in local population adaptation.

## 2 MATERIALS AND METHODS

### 2.1 Generation of chimeric pTAS2R20 receptors

**FIGURE 1:**
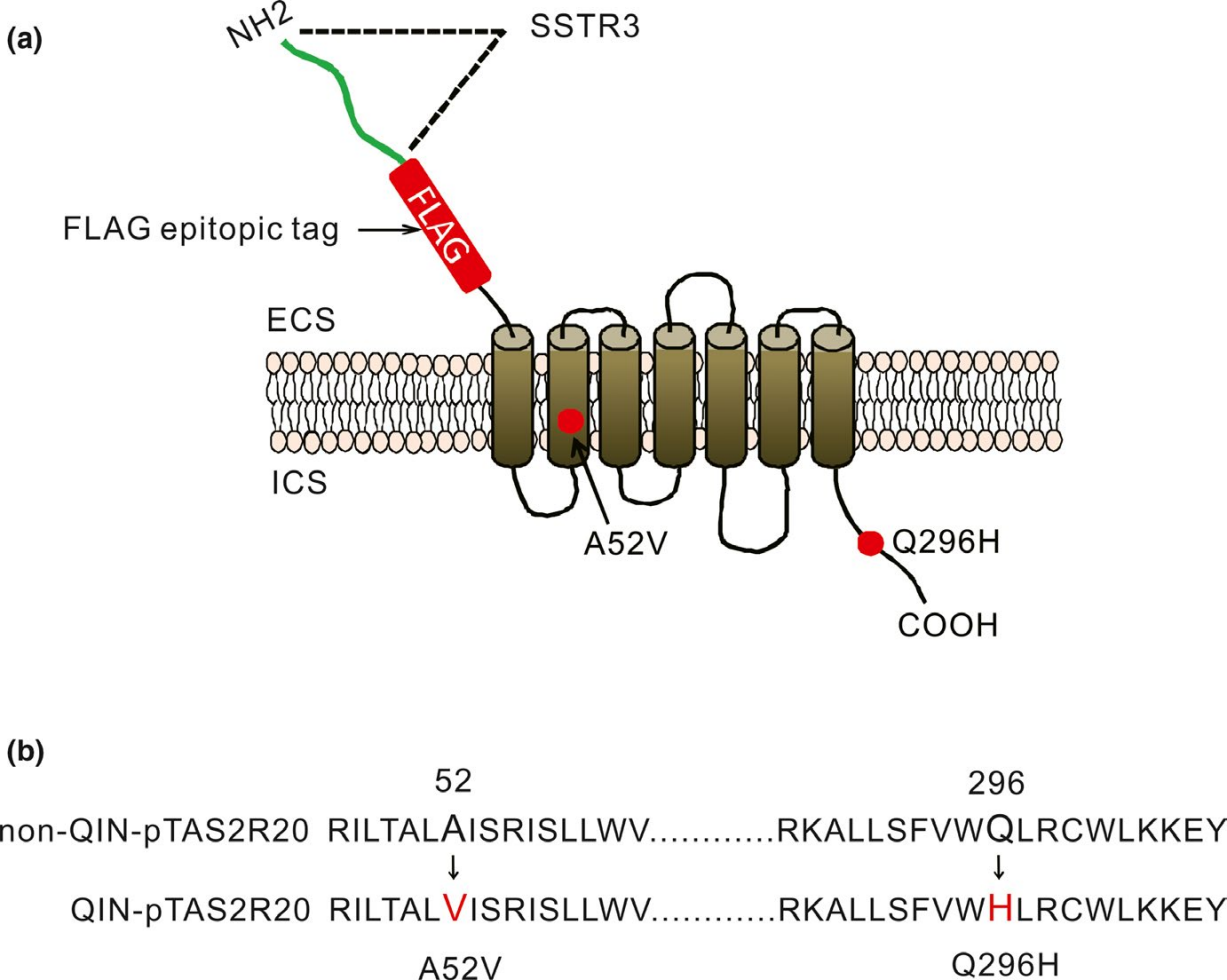
Schematic representation of the structure of pTAS2R20 for functional experiments and amino acid sequence of SNP variations. (a) Amino acids mutated in Qinling pandas are shown as red dots. The FLAG epitopic tag was used to detect protein expression by immunohistochemistry, which is surrounded by the red box. Forty‐five amino acids of rat SSTR3, used as a cell surface‐targeting signal, are indicated with a green wavy line. (b) The two amino acid mutation sites of pTAS2R20. The red‐labeled amino acids are mutated positions 52 and 296 of the Qinling pandas’ pTAS2R20 amino acid sequence

The pTAS2R20 mutants (Qinling) were generated from the Sichuan template (pTAS2R20‐AQ), using the Agilent QuikChange Lightning Site‐Directed Mutagenesis Kit (Catalogue #210518). Manufacturer's instruction was followed and the primers used for the site‐directed mutagenesis were A52V_for 5′‐CTGCTCTGGCGATCTCCAG‐3′ and A52V_rev 5′‐CTGGAGATCGCCAGAGCAG‐3′, Q296H_for 5′‐CGTGTGGCAGCTGAGATGC‐3′ and Q296H_rev 5′‐GCA TCTCAGCTGCCACACG‐3′. The coding region of p*TAS2R20* was cloned into the pcDNA3.1 vector (Invitrogen) and amino terminally tagged with 45 amino acids of rat SSTR3 as a cell surface targeting signal (Masataka & Takumi, 2018) followed by FLAG tag (DYKDDDDK) (Figure 1a), which dose not interfere with the signaling of heptahelical receptors and can be used for immunocytochemistry analysis (Coin et al., 2013). The two directionally selected nonsynonymous sites, A52V and Q296H, of pTAS2R20 were indicated red (Figure 1b). The four pTAS2R20 variants were functionally expressed in HEK‐293T cells (Pronin et al., 2007). We reconstituted the GPCR reaction system, and mG15, a gift from Dr. Stephen Libeles of Harvard Medical School was used as an effector (Bufe et al., 2002) which is necessary for GPCR signal transduction. G‐CaMP was generously provided by Prof. Loren L. Looger (Howard Hughes Medical Institute, Janelia Farm Research Campus) as a fluorescent indicator (Marella et al., 2006). The changes in fluorescence were observed by fluorescence microscopy and measured with ImageJ.

### 2.2 Immunocytochemistry

The transfected HEK‐293T cells were seeded onto poly‐L‐lysine‐treated coverslips 24 hr after transfection and washed with PBS three times, then fixed with 4% paraformaldehyde at room temperature for 15 min. To reduce nonspecific binding, the cells were incubated in 4% bovine serum albumin for 1 hr. The first anti‐FLAG antibody (1:1,000, 8146S, Cell Signaling) was added to detect the expression of the receptors, followed by incubation overnight at 4°C. The plasma membrane localization marker protein fused with GFP (mGFP) was used to label the cell membrane. After washing five times, the coverslips were incubated in a dilute solution of the fluorescent secondary antibody goat anti mouse IgG (1:500, ab150116, Abcam), usually for a few hours at room temperature (Singh, Pydi, & Upadhyaya, 2011). Photomicrographs were taken with a Leica TCS SP2 Laser Scan Inverted microscope.

### 2.3 Determination of quercitrin contents in bamboo

*Bashania fargesii* and *F. qinlingensis* samples were collected from the Qinling Mountains, and *B. faberi* and *F. denudata* samples were collected from the Qionglai and Minshan Mountains. Perennial bamboo leaves were freeze‐dried and crushed. The purity of the quercitrin (Sigma‐Aldrich) used as the standard sample was >98%. A 50 mg sample was added to 1 ml methanol, and extraction was performed at 65°C for 4 hr. The LC‐ESI‐MS analysis method was used in this experiment. A ultra high‐performance liquid chromatography system was used in the chromatographic system. According to the properties of flavonoids, the reaction conditions were as described previously (Wu et al., 2010). The external standard method was used for quantitative analysis with three repetitions of each sample, and the fitting curve was prepared by using different concentrations of standard samples. The dose–response curves were corrected for background fluorescence and normalized to the maximal response observed.

### 2.4 pTAS2R20 functional assay

HEK‐293T cells were seeded at a density of 3 × 10^3^ cells per well into 96‐well black‐wall, clear‐bottom microtiter plates (Corning). After 24 hr of adherent culture, the vectors of the bitter receptor, mG15, and G‐CaMP were transiently cotransfected into HEK‐293T cells (Behrens, Blank, & Meyerhof, 2017). After 36 hr, the cells were stimulated with the optimal activation concentration of bitter compounds dissolved in serum‐free medium and DMSO, not exceeding a final concentration of 1% (v/v). The activation of the bitter receptors led to the release of Ca^2+^ from the endoplasmic reticulum and an increase in the concentration of cytoplasmic Ca^2+^ which was detected with the calcium indicator G‐CaMP. Fluorescence images were recorded every 3 s within a period of 30 s before and 120 s after the addition of bitter compounds. The fluorescence values of the cells at each time point were measured with ImageJ software. We counted at least 20 cells in every group and calculated the changes in their fluorescence values compared with the initial stage. The response curves were corrected according to the background fluorescence and normalized to the maximal response observed. The formula for calculating the ratio of the cell fluorescence increase compared with the initial value was as follows:

$$F=\frac{F_{X}-F_{\text{CX}}}{F_{0}-F_{\text{C0}}},$$

where *F* is the fold change of the fluorescence value, *F*_0_ is the average fluorescence intensity of the cells before adding the agonists at the first three time points, *F*_C0_ and *F*_CX_ are the background fluorescence values, and *F*_x_ is the fluorescence intensity of the cells recorded every 3 s.

## 3 RESULTS

**FIGURE 2:**
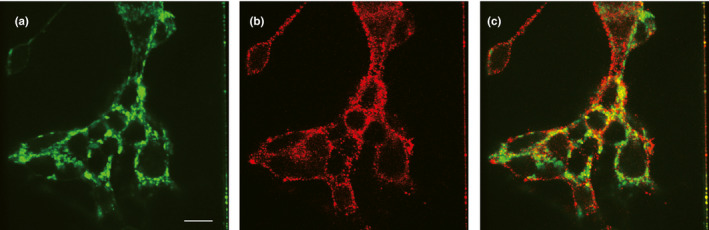
Immunocytochemical detection of pTAS2R20 at the cell surface. (a) The cell surface is labeled by a plasma membrane protein fused with GFP (mGFP). (b) The amino terminus of pTAS2R20 is tagged with FLAG (DYKDDDDK), and the FLAG epitope is detected by an anti‐FLAG primary antibody and an Alexa561‐conjugated secondary antibody. (c) Overlap of (a) and (b). Scale bar = 10 μm

p*TAS2R20* was cotransfected into HEK‐293T cells with the plasma membrane localization marker protein fused with GFP (mGFP) to determine whether pTAS2R20 was successfully localized at the cell membrane. Then, the cell membrane was visualized by using mGFP (green fluorescence, Figure 2a). The immunocytochemical detection of pTAS2R20 (red fluorescence, Figure 2b) showed that pTAS2R20 localized to the cell membrane. Superimposed red and green fluorescence appeared yellow, which indicated that the two markers colocalized at the cell membrane (Figure 2c). Henceforth, pTAS2R20‐expressing cells were stimulated with several common bitter compounds and bamboo‐derived bitter chemicals. The results showed that all four pTAS2R20 variants were specifically responsive to quercitrin.

**FIGURE 3:**
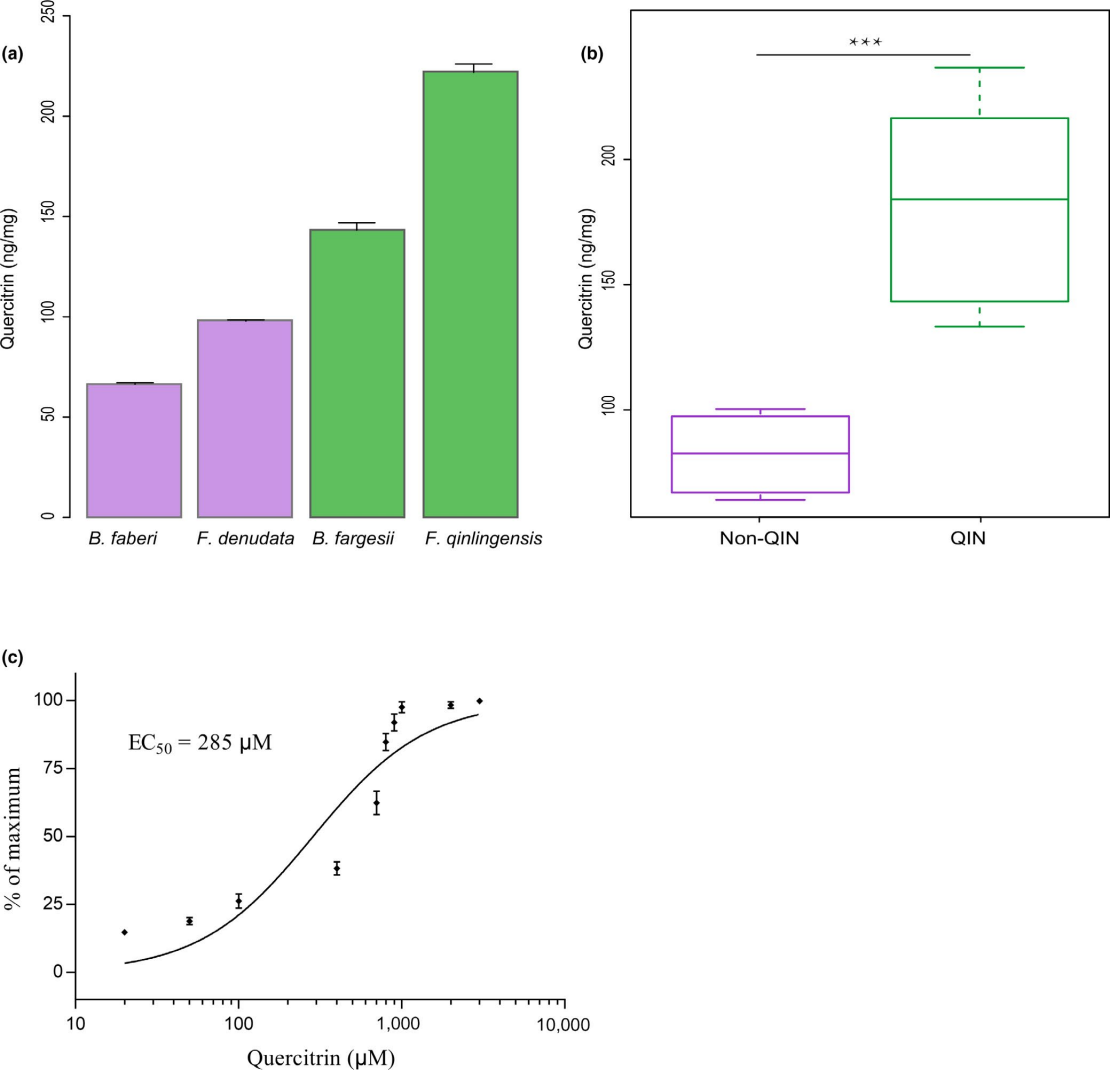
The contents of quercitrin in bamboo leaves and dose–response curves of quercitrin. (a) Contents of quercitrin in representative samples of the giant panda staple food of bamboo leaves from Qinling Mountains (QIN) and other areas (non‐QIN). *B. fargesii* and *F. qinlingensis* were collected in the Qinling Mountain (QIN). *F. denudata* and *B. faberi* were collected in other areas (Qionglai and Minshan Mountains). (b) Significant difference analysis of quercitrin content in bamboo leaves of QIN and non‐QIN areas. (c) Dose–response curves of quercitrin in the activation of pTAS2R20. EC_50_ = 285 μM

We quantified the contents of quercitrin in the leaves of *B. fargesii* and *F. qinlingensis*, consumed in the diet of Qinling pandas, and in the leaves of *F. denudate* and *B. faberi*, consumed in the diet of pandas from other areas. The quercitrin content of *F. qinlingensis* leaves was the highest among the four examined bamboo species, reaching 222.3 ng/mg, followed by that of *B. fargesii* at 143.7 ng/mg. In contrast, the quercitrin contents quantified in *F. denudata* and *B. faberi* leaves were 98.2 and 66.4 ng/mg, respectively, which are much lower than those of the two bamboo species sampled in the Qinling Mountains (*p* < 0.05) (Figure 3a, b).

To determine the optimum concentration of quercitrin required to activate pTAS2R20, we measured the highest potency of quercitrin, which activated pTAS2R20 at a low molar concentration, in a dose–response curve. When the concentration of quercitrin was increased, the activation of pTAS2R20 was demonstrated by a sigmoidal curve in which the concentration resulting in 50% of maximal effect (EC50) was 285 μM, and maximum activation occurred at a concentration of several thousand micromolar (Figure 3c).

**FIGURE 4:**
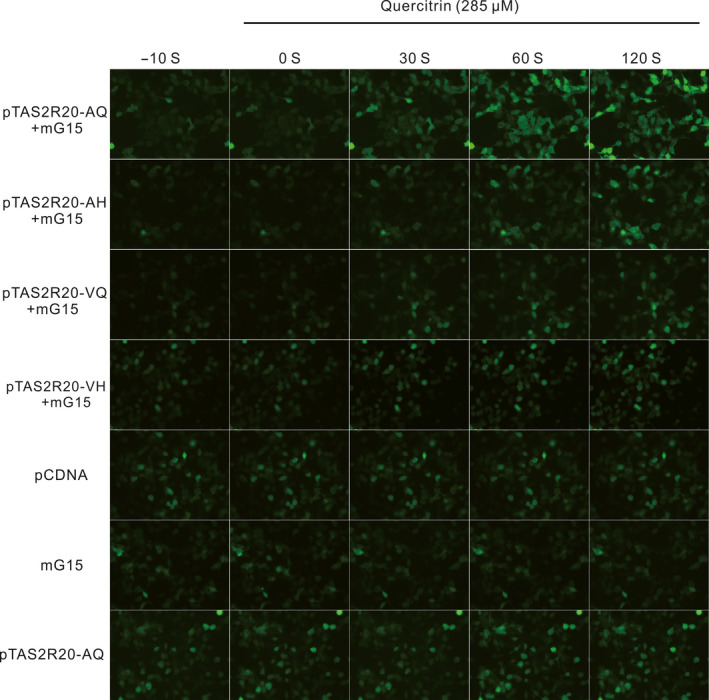
Ca^2+^ fluorescence images of cells before and after treatment with quercitrin at five time points. Quercitrin was added at the 0 s time point. The control groups (pcDNA, mG15, pTAS2R20) did not receive mG15, and they did not respond to the agonist (quercitrin, 285 μM), indicating that both the receptor (pTAS2R20) and signaling molecule (mG15) are necessary for the activation of the bitter taste receptors

**FIGURE 5:**
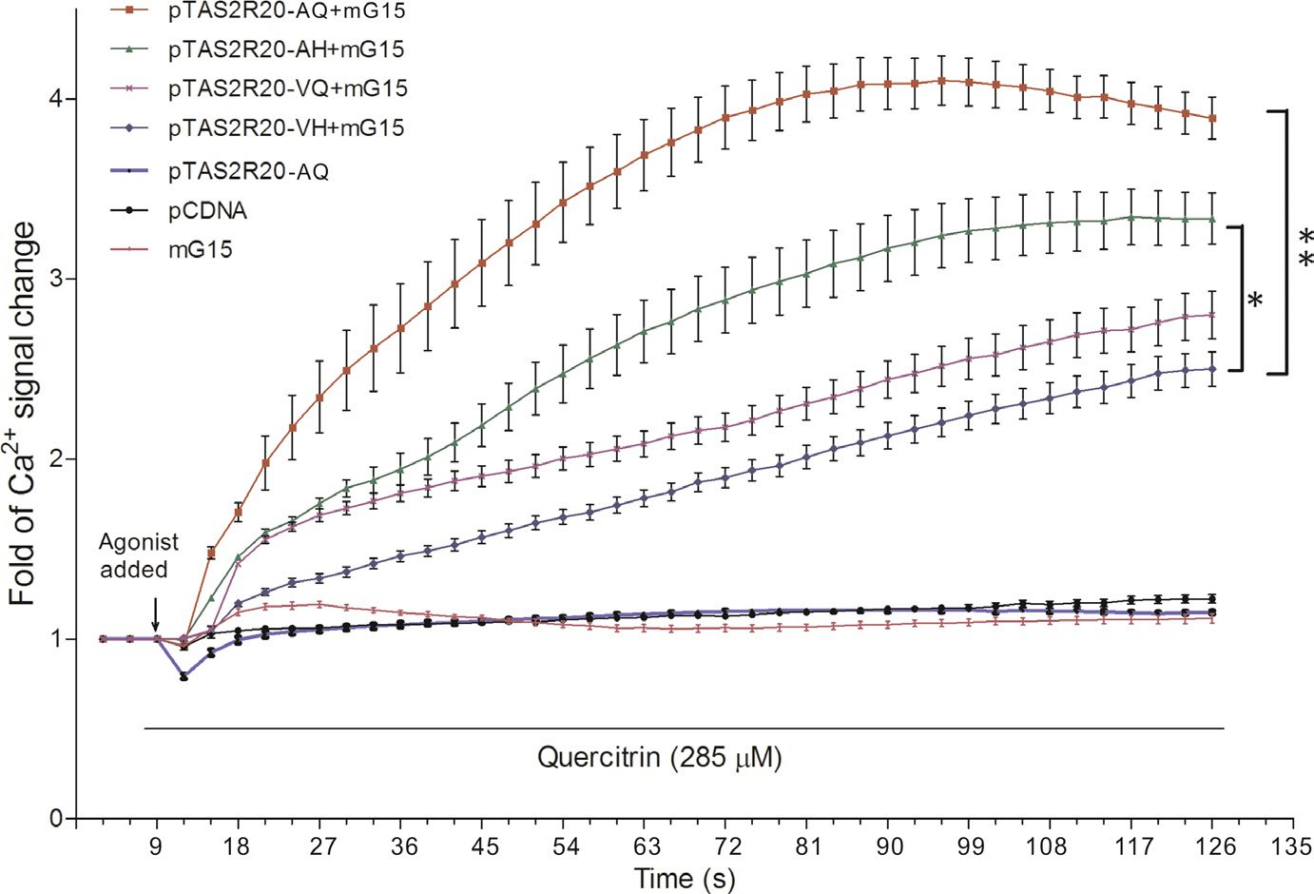
Fold change of the Ca^2+^ change after quercitrin treatment. Each line represents the change in the Ca^2+^ fluorescence signal in more than 20 representative single cells in each group during the time course. The common pTAS2R20 is mostly found in pandas outside of the Qinling Mountains; The pTAS2R20‐AH variant carries the mutant site Q296H, and the pTAS2R20‐VQ variant carries the mutant site A52V. These two variants are laboratory‐produced types; The pTAS2R20‐VH variant carries two mutant sites at A52V and Q296H, which are mostly found in Qinling pandas; pcDNA is the vector control; and mG15 is the signaling molecule control

To characterize the effect of these two directionally selected nonsynonymous sites on the function of pTAS2R20 in the response to quercitrin, we activated each pTAS2R20 variant with the optimal activation concentration of quercitrin. Cell fluorescence images obtained at five time points before and after quercitrin treatment are shown in Figure 4. The fluorescence intensity of the cells expressing pTAS2R20 significantly increased in comparison with that in the control groups (pcDNA, pTAS2R20, mG15), which showed no significant change. This confirmed that pTAS2R20 expressed in HEK‐293T cells was responsive to quercitrin. Therefore, the experimental group harboring the pTAS2R20‐AQ variant exhibited the strongest reaction, whereas the group with the pTAS2R20‐VH variant showed the weakest reaction. Overall, the order of the reaction activities of the experimental groups was pTAS2R20‐AQ > pTAS2R20‐AH > pTAS2R20‐VQ > pTAS2R20‐VH. To further quantify the differences in the responses of different pTAS2R20 variants to quercitrin molecules, we quantified the fluorescence intensity of representative single cells in each group at 42 time points by using ImageJ (Figure 5). The statistical results showed that the maximum value of fold of Ca^2+^ signal change in the VQ mutation group was 2.80, and those in the other groups were as follows: AH 3.34, VH 2.50, and pTAS2R20‐AQ 4.10. These results consistently suggested that the two nonsynonymous sites, A52V and Q296H, greatly reduced the sensitivity of the receptor in the response to quercitrin in bamboo (Figure 5).

## 4 DISCUSSION

In this study, we showed that two directionally selected nonsynonymous sites, A52V and Q296H, are associated with decreased sensitivity to quercitrin in Qinling pandas, which confers upon the pandas a dietary preference for high‐quercitrin bamboo leaves. In agreement with the hypothesis that polymorphisms in sensory receptor genes may alter perception by encoding functionally distinct receptor types (Bufe et al., 2005; Wooding et al., 2006), we add new evidence that genetic variations in a bitter receptor gene (p*TAS2R20*) are correlated with differences in bitterness (quercitrin) recognition. Bitter taste perception provides an important means for animals to detect bitter compounds in the environments that they occupy. As animals explore new environments and diets, they may encounter distinct bitterness, which imposes selective pressure on their bitter taste receptor genes, leading to individual differences in bitter taste sensitivity (Li & Zhang, 2013; Shan et al., 2018; Shi, Zhang, Yang, & Zhang, 2003; Wooding et al., 2006). The TAS2R genes of the giant panda are expected to have experienced selective pressure during the course of the dietary shift of pandas from carnivorous to omnivorous to herbivorous and, finally, their specialization for bamboo consumption. Our previous study demonstrated that the giant and red pandas harbor more putative functional bitter taste receptors than other carnivores, and several of their TAS2R genes seem to have experienced selective pressure, as these species are challenged with many more bitter compounds in diets (Shan et al., 2018). Although natural selective signatures on several TAS2R genes in the pandas have been revealed, the functional effects of the selective pressure on bitter taste perception have not been examined. The present study has provided a fine example of the effects of directional selection on giant panda dietary preferences, providing direct evidence of a close correlation between taste function and the pandas’ feeding ecology.

pTAS2R20 was shown to be specifically activated by quercitrin, a flavonoid monomer found in various plants including bamboos that have previously been identified as the agonist of a bitterness receptor of the herbivorous insect *Papilio hospiton* (Sollai, Barbarossa, Solari, & Crnjar, 2015). Studies in humans have suggested that bitter taste receptors are usually broadly tuned to recognize numerous compounds possessing common chemical groups responsible for mediating receptor‐agonist interactions (Bufe et al., 2005; Meyerhof et al., 2010). Given that a limited number of bitter compounds were assayed here and that bamboo produces flavone compounds, the actual number of dietary agonists for TAS2R20 may be greater. However, the two directionally selected sites, A52V and Q296H, indeed conferred TAS2R20 with a significantly decreased sensitivity to quercitrin, which may reduce aversion to quercitrin in Qinling pandas. Consequently, although higher quercitrin was contained in the bamboo leaves from *B. fargesii* and *F. qinlingensis* in Qinling Mountains, Qinling pandas taste the leaves better than other pandas taste the bamboo leaves from *F. denudate* and *B. faberi* in other areas. Therefore, the two directionally selected nonsynonymous sites may explain why Qinling pandas prefer to consume more bamboo leaves than other pandas do.

Recent studies on chemical substances in the giant panda staple food bamboo have focused on nutrition and minerals (Christian et al., 2015; Nie, Zhang, et al., 2015), but there are few systematic reports of bitter substances. Thus, we screened agonists for pTAS2R20 from a limited number of common chemicals found in bamboos and other plants. According to previous studies indicating that giant pandas are inclined to ingest bamboos that contain less tannin (Hansen et al., 2010; Zhao, Liu, & Ma, 2001), we listed quercitrin and tannin as important potential agonists of the receptor. Here, functional expression experiments confirmed that quercitrin is an agonist of pTAS2R20 that may play a significant role in feeding preference. Although tannin is the correct agonist, as we previously conjectured (Zhao et al., 2013), this result is not surprising, since giant pandas possess numerous bitter receptors, and some of them may specifically recognize tannic acid.

The giant panda belongs to the order Carnivora (Wei et al., 2014); nevertheless, it has evolved certain characteristics of herbivores including a skull, jaw musculature and dentition that are suitable for a fibrous diet and a pseudothumb that is specifically used for handling bamboo (Hu et al., 2017; Zhang, Pan, Li, Oxnard, & Wei, 2007). Mutations in *DUOX2* led to a decrease in thyroxine levels, which helped to reduce energy consumption for adaptation to a low‐energy bamboo diet (Nie, Speakman, et al., 2015). These studies have emphasized behavioral, physiological, morphological, and genetic adaptations. However, it is rare to reveal the molecular basis of feeding adaptation in giant pandas from the perspective of bitterness receptors function, with the exception of similar reports on the pseudogenization of the umami taste receptor gene TAS1R1, which is associated with eating meat (Hu et al., 2017). This research fills a gap in explaining the evolution of diet adaptation from the perspective of the molecular function of bitterness receptors, especially in nonmodel animals. This study is also a typical example for linking genome‐scan candidate gene to ecological adaptation at functional verification level.

## CONFLICT OF INTEREST

The authors declare that they have no conflict interest.

## AUTHOR CONTRIBUTION

**Xiangxu Hu:** Data curation (equal); Investigation (equal); Methodology (equal). **Guan Wang:** Data curation (equal); Investigation (equal); Methodology (equal). **Lei Shan:** Writing‐original draft (equal); Writing‐review & editing (equal). **Shuyan Sun:** Data curation (supporting); Investigation (supporting); Methodology (supporting). **Yibo Hu:** Writing‐original draft (equal); Writing‐review & editing (equal). **Fuwen Wei:** Conceptualization (lead); Project administration (lead). 

## Data Availability

The data that support the findings of this study have been deposited in Dryad with https://doi.org/10.5061/dryad.wh70rxwjv.
